# Multifaceted mechanisms and targeted delivery of mesenchymal stem cell-derived exosomes in cardiovascular diseases: a translational medicine perspective

**DOI:** 10.3389/fmed.2025.1729698

**Published:** 2025-12-04

**Authors:** Xiao Jin, Zongjun Liu

**Affiliations:** Cardiology Department, Putuo Hospital, Shanghai University of Traditional Chinese Medicine, Shanghai, China

**Keywords:** MSC-Exo, myocardial infarction, myocardial ischemia-reperfusion injury, atherosclerosis, heart failure, cardiomyopathy, myocarditis, pulmonary hypertension

## Abstract

As cardiovascular disease (CVD) is the leading cause of death worldwide, its prevention and treatment is urgent and new therapeutic targets are urgently needed. Mesenchymal stem cell-derived exosomes (MSC-Exo) have emerged as promising nanotherapeutics due to their regenerative capacity, low immunogenicity, and multilineage differentiation potential. This review systematically integrates the therapeutic mechanisms of MSC-Exo in seven major cardiovascular indications, including myocardial infarction, myocardial ischemia–reperfusion injury, atherosclerosis, heart failure, cardiomyopathy, myocarditis, and pulmonary hypertension, while exploring advanced engineering strategies to enhance its efficacy. Through comprehensive analysis of current preclinical studies, we demonstrated that MSC-Exo mainly exerts cardioprotective effects by promoting angiogenesis, inhibiting apoptosis, fibrosis, inhibiting inflammatory responses, and regulating immune responses. In addition, we also highlight innovative delivery methods, including intrapericardial administration for immunomodulation, ischemic myocardium-targeting peptides, and functional hydrogel encapsulation that significantly improve exosome retention and bioavailability. The fusion of biological mechanisms and engineering solutions makes MSC-Exo a multifunctional platform for cardiovascular regenerative medicine, with emerging clinical translation potential through optimized delivery systems and precise targeting strategies, in order to provide new ideas for the treatment of cardiovascular diseases with MSC-Exo.

## Graphical abstract

**Figure:**
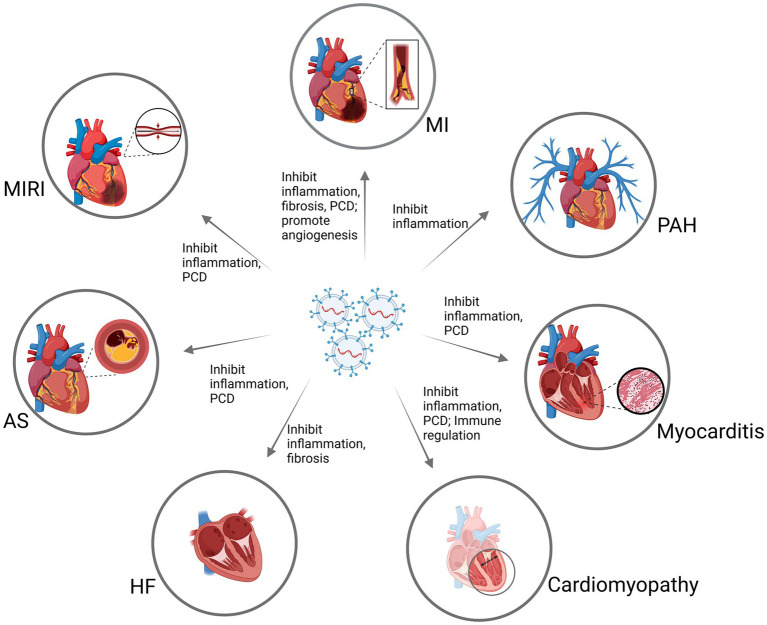

## 1 Introduction

In the 2023 World Heart Report released by the World Heart Federation (WHF), it is pointed out that cardiovascular diseases have been the leading cause of death globally for decades. In 2019, the number of deaths from cardiovascular diseases was 18.6 million, accounting for 33% of the total global deaths. By 2021, the number of deaths from cardiovascular diseases had exceeded 20 million (1). Therefore, the prevention and treatment of cardiovascular diseases are urgent, and new therapeutic targets are needed.

Stem cells are a type of multipotent cells with self-replication ability that can differentiate into various types of cells under specific conditions, possessing tissue repair and regeneration capabilities (2). They can be broadly classified into three categories: embryonic stem cells, adult stem cells, and induced pluripotent stem cells. Adult stem cells include hematopoietic stem cells (HSC), mesenchymal stem cells (MSC), neural stem cells (NSC), and endothelial stem/progenitor cells (EPC) (3). As a therapeutic approach in regenerative medicine, stem cells have received widespread attention and provide strong support, especially in the treatment of cardiovascular diseases (4). Among them, MSCs have garnered significant attention due to their high proliferative activity, low immunogenicity, multilineage differentiation potential, and relatively fewer ethical constraints compared to other types of stem cells (5). However, the potential of stem cell therapy to promote tumor growth and metastasis remains a concern in the field of regenerative medicine (5).

Exosomes (Exo) are tiny vesicles encapsulated by a lipid bilayer, typically with a diameter of 40–100 nm. They can be produced by almost all types of normal cells and are widely present in biological fluids such as saliva, plasma, urine, ascites, and bile (6). Exo are considered micromodel of their parent cells, partly because they carry a unique set of biomolecules from the parent cells (7), but without the side effects associated with the parent cells (8). Therefore, exosome therapy derived from stem cells is regarded as a safer and more effective alternative to stem cell therapy, providing similar clinical effects without the biosafety issues related to live cell transplantation (8, 9). Among them, exosomes derived from mesenchymal stem cells (MSC-Exo) have received the most attention. Studies have shown that MSC-Exo can promote apoptosis (10), remodel blood vessels (11), and exert anti-inflammatory effects (12), making them an effective means of treating cardiovascular diseases.

This article reviews the treatment of multiple cardiovascular diseases with MSC-Exo, and discusses the biological characteristics of MSC-Exo. This study aims to explore the mechanism of MSC-Exo in the treatment of different cardiovascular diseases. Different from previous studies, which mainly started from a certain cardiovascular disease, this paper summarizes existing articles and systematically explains the effects of MSC-Exo in different cardiovascular diseases.[Including myocardial infarction (MI), myocardial ischemia reperfusion (MIRI), atherosclerosis (AS), heart failure (HF), cardiomyopathy, myocarditis, pulmonary arterial hypertension (PAH)]. Therefore, it can more comprehensively reveal the complexity of MSC-Exo in treating cardiovascular diseases. In addition, this study also explores new ways to increase the pharmacological effects of MSC-Exo, in order to provide new ideas for MSC-Exo in the treatment of various cardiovascular diseases.

## 2 MSC-Exo

Exo are biological nanoscale spherical lipid bilayer vesicle secreted by cells, with a diameter of about 40-100 nm, which can be secreted by almost all cells (13). In 1981, Trams et al. first discovered vesicles in normal cells and tumor cells, and it was found that they may have physiological functions (14). In 1983, Johnstone et al. observed the secretion of sheep reticulocyte vesicles under an electron microscope and first discovered Exo in the supernatant of sheep red blood cells cultured *in vitro*. At that time, researchers believed that this membrane-structured cell vesicle could carry unnecessary proteins from one cell to another during cell growth and development, and thus concluded that this vesicle was the “disposal” of cell metabolism (15). With the in-depth study of Exo, the function of Exo was gradually discovered. In 1996, the Raposo team discovered that Exo has a role in antigen presentation *in vivo* (16). In 2007, Johnstone et al. proposed that Exo can exchange genetic material between mast cell (17). Since then, Exo have gradually attracted widespread public attention.

**Figure 1:**
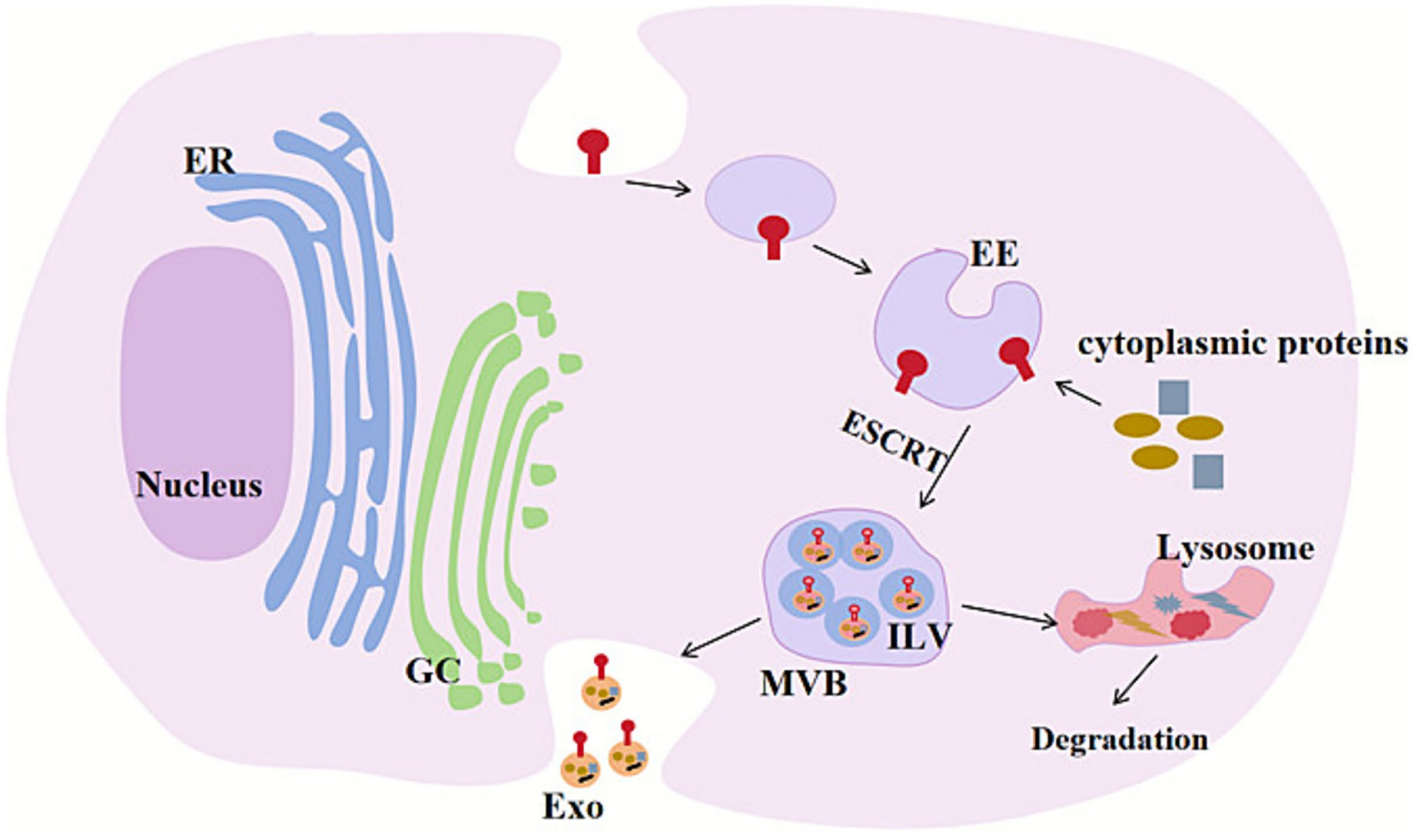
The production process of Exo. The plasma membrane is depressed inward or the lysosomal particles in the cells are invaginated, forming multiple endocytoses. These individuals will merge with each other to form EE. They will further sag inward, enveloping substances such as protein and lipids, and forming multiple ILVs. With the formation and increase of vesicles in the cavity, EE gradually transformed into late endosome, also known as MVB. MVB further matured in cells, transferred to plasma membrane and fused with plasma membrane. This fusion process requires the participation of many regulatory molecules, such as ESCRT complex. During the fusion process, ILV is released into extracellular body fluids, and these released vesicles are called Exo. In addition, a part of MVB will be transported to the vicinity of lysosomes, fused with lysosomes and subjected to degradation. ER, endoplasmic reticulum; GC, Golgi complex; EE, early endosome; ESCRT, Endosomal Sorting Complexes Required for Transport; Exo, Exosomes; ILV, Intraluminal Vesicles; MVB, Multivesicular bodies.

Extracellular vesicles (EVs) are a heterogeneous group of membrane structures that include exosomes (Exo), microvesicles, and apoptotic bodies (18). Unlike the formation mechanisms of the other two subgroups, Exo are generated through the inward budding of the plasma membrane (19). The plasma membrane invaginates in specific regions to form early endosomes (EE), which gradually accumulate cargo molecules during maturation and undergo morphological changes to become late endosomes or multivesicular bodies (MVB) (20), MVBs contain multiple intraluminal vesicles (ILVs) (21), which are formed by the inward budding of the endosomal membrane and contain specific proteins, lipids, nucleic acids, and other molecules. Subsequently, some MVBs are transported near lysosomes and fuse with them for degradation. Another portion of MVBs fuse with the plasma membrane to release ILVs, generating Exo (22) (Figure 1).

Exo contain a variety of biological components, such as proteins, lipids, RNA, DNA, enzymes, transcription factor receptors, and other bioactive substances (23). The proteins can be divided into two types: one type is common to all Exo, including glycosylphosphatidylinositol (GPI)-anchored proteins, tetraspanins (cluster of differentiation (CD) 81 and 82, CD9, CD63), heat shock proteins and chaperones (HSP20, HSP60, HSP70, HSP90), ESCRT complexes, proteins related to membrane trafficking and fusion (ADP-ribosylation factor (ARF), Rab proteins, annexins), integrins, and so on (6, 24). The other is cell-type specific, representing a unique set of biomolecules that serve as a “molecular signature” of their parent cells. It is precisely this source-dependent cargo that governs the functional diversity of exosomes, which is why this review focuses on the biological characteristics, functions, and mechanisms of mesenchymal stem cell-derived exosomes (MSC-Exo) in various cardiovascular diseases.

## 3 Biological characteristics of MSC-Exo

MSC-Exo have multiple sources, primarily derived from bone marrow, adipose tissue, and umbilical cord. They demonstrate potent abilities in repairing tissue damage through paracrine effects, including in the cardiovascular system (25), respiratory system (26), nervous system (27), endocrine system (28), digestive system (29), liver injury (30), skin injury (31), osteoarthritis (32) and other diseases. MSC-Exo delivered to the heart of MI rats by intraperitoneal injection can inhibit the transformation of myofibroblasts, inhibit cardiomyocyte apoptosis, promote angiogenesis, inhibit inflammatory response, etc., which can improve the cardiac function of MI rats (33). In the treatment of pneumonia, it works through its anti-inflammatory and immunomodulatory effects and ability to induce tissue regeneration (34). It can reduce neuroinflammation, promote neovascularization, induce neurogenesis, and reduce apoptotic loss of neurons through neuroprotective and immunomodulatory microRNAs, nerve growth factors, and anti-inflammatory cytokines (35). Inhibition of Hedgehog/Smoothened pathway reduces epithelial-mesenchymal transition and renal fibrosis in patients with diabetic nephropathy (36). Pancreatic tissue was collected 7 days after intravenous injection of MSC-Exo treatment of diabetic mice. The results showed that upregulation of genes related to tissue regeneration pathways in pancreatic tissue increased the expression of genes, improved islet function, and promoted pancreatic regeneration (37). Furthermore, they promote the repair of damaged substructures, including the regeneration of blood vessels, nerves, and hair follicles, promote macrophage polarization, wound angiogenesis, cell proliferation, and cell migration by influencing angiogenesis-related and antifibrotic pathways, and inhibit scar formation by inhibiting the overproduction of extracellular matrix (38). In summary, MSC-Exo possess anti-inflammatory, immunomodulatory, angiogenesis-promoting, and apoptosis-inhibiting functions, making them of great significance in the treatment of various diseases.

Furthermore, MSC-Exo possess the ability to cross immune barriers, exhibit high biocompatibility, enhanced stability, and low immunogenicity, allowing them to transport functional cargos to specific cells. As such, they are often utilized as drug carriers (39, 40). Functional molecules such as overexpressed MicroRNA (miRNA), long non-coding RNA (lncRNA), small interfering RNA (siRNA), DNA, proteins, and other substances can be encapsulated within MSC-Exo, or drugs can be co-incubated with parent cells to generate drug-containing MSC-Exo, which are then delivered to specific sites to exert their effects (41, 42). Currently, engineered Exo that achieve efficient drug delivery and high targeting specificity hold greater potential. These are modified through genetic, physical, or chemical methods to fulfill specific functions (43). Physically induced drug loading involves mechanical or physical disruption through external forces, such as electroporation, sonication, freeze–thaw cycles, and extrusion. Chemically induced drug loading utilizes saponins or transfection reagents to bypass the Exo membrane (44). These techniques collectively establish the important role of MSC-Exo in the treatment of cardiovascular diseases (Table 1).

**Table 1:** Summary of MSC-Exo in the treatment of cardiovascular diseases.

Disease model	Key biological effects	Key molecules/MiRNAs involved (References)	Core signaling pathways/targets
Myocardial infarction (MI)	Promotes angiogenesis, inhibits apoptosis/fibrosis/inflammation, improves cardiac function	miR-132 (49), miR-543 (48), miR-21a-5p (60), miR-24-3p (90), miR-183-5p (101)	RASA1, COL4A1, PDCD4/PTEN, NF-κB, HMGB1
Myocardial ischemia–reperfusion injury (MIRI)	Inhibits apoptosis/pyroptosis/ferroptosis, reduces oxidative stress, regulates autophagy	miR-125a-5p (53), miR-183-5p (63), miR-455-3p (72), Mir9-3hg (77)	DAAM1, FOXO1, MEKK1, Pum2/PRDX6
Atherosclerosis (AS)	Reduces plaque formation, promotes M2 polarization, inhibits inflammation	miR-21a-5p (93), miR-let7 (94), miR-100-5p (95), mir-28 (116)	KLF6, HMGA2/NF-κB, FZD5/Wnt/β-catenin, TEAD1
Heart failure (HF)	Inhibits cardiomyocyte apoptosis/hypertrophy/fibrosis, improves cardiac remodeling	miR-30e (122), miR-129-5p (124)	LOX1/NF-κB, TRAF3/NF-κB,
Cardiomyopathy	Suppresses inflammation, ameliorates cardiomyocyte senescence and mitochondrial dysfunction	miR-9-5p (129)	VPO1/ERK
Myocarditis	Inhibits virus-induced apoptosis and ferroptosis, modulates autophagy	let-7a-5p (135)	SMAD2/ZFP36
Pulmonary arterial hypertension (PAH)	Inhibits pulmonary vascular remodeling, improves mitochondrial function, promotes M2 polarization	miR-17/miR-204 (137)	STAT3

## 4 General effects and mechanisms of MSC-Exo in cardiovascular diseases

**Figure 2:**
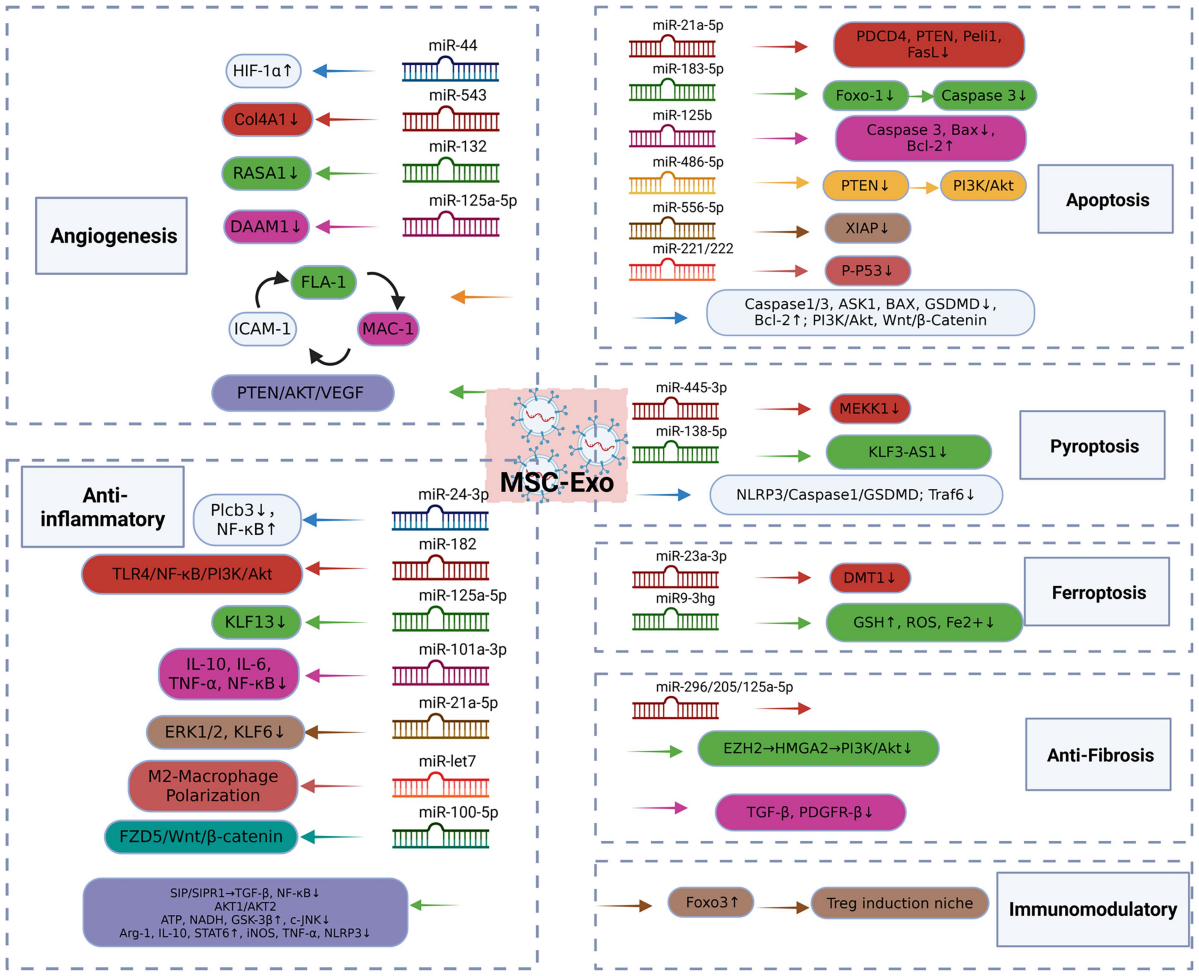
MSC-Exo mechanism of action. MSC-Exo can significantly enhance the tube formation of endothelial cells, up-regulate the expression level of angiogenic factors, accelerate angiogenesis, and exert myocardial protection by inhibiting apoptosis, reducing autophagy flux, and inhibiting pyroptosis and ferroptosis. It can also regulate the renin-angiotensin system, accelerate the transformation of angiotensin II, improve cardiac remodeling, block the activation in fibroblasts and inhibit fibrosis. In addition, it plays an anti-inflammatory role by promoting M2 polarization of macrophages and inhibiting the expression of inflammatory factors. And target the immune system to increase the proportion of Treg cells and maintain the immune homeostasis of the body. MSC, Mesenchymal Stem Cell; Exo, Exosomes; HIF-1α, Hypoxiainduciblefactor-1α; COL4A1, Collagen Type IV Alpha 1 Chain; RASA1, RASp21 protein activator 1; MAC-1, Macrophage-1 antigen, FLA-1, Lymphocyte Function-Associated Antigen 1, ICAM-1, Intercellular Adhesion Molecule-1; ASK1, Apoptosis signal-regulating kinase1; Bcl-2, B-cell lymphoma-2; LOX-1, Oxidized Low Density Lipoprotein Receptor 1; GSDMD, Gasdermin D; BAX, Bcl-2-associated X protein; PDCD4, Programmed Cell Death Protein 4; PTEN, Phosphatase and Tensin Homolog; Peli1, Pellino Homolog 1; FasL, Fas Ligand; Foxo1, Forkheadbox transcription factor O1; PI3K, phosphatidylinositol 3-kinase; XIAP, X-linked inhibitor of apoptosis protein; MEKK1, Mitogen-Activated Protein Kinase Kinase Kinase 1; KLF3-AS1, KLF3 Antisense RNA 1; Traf6, TNF receptor associated factor 6; DMT1, Divalent metal transporter 1; GSH, Glutathione; ROS: reactive oxygen species; EZH2, Enhancer of zeste homolog 2; HMGA2, High mobility group AT-hook 2; TGF-β, Transforming Growth Factor-β1; PDGFR1, platelet-derived growth factor receptor-β; Plcb3, Phospholipase C-Beta3; TLR4, Toll-like receptor 4; ERK1/2, Extracellular signal-regulated protein kinase 1/2; KLF6, Kruppel-like factor 6; FZD5, Frizzled 5; SIP, sphingosine 1-phosphate; ATP, Adenosine Triphosphate; NADH, Nicotinamide Adenine Dinucleotide; GSK-3β, Glycogen synthase kinase-3β; c-JNK, c-JunN -terminal kinase; Arg1, Arginase-1; iNOS, Inducible nitric oxide synthase; STAT6, Signal Transducer and Activator of Transcription 6.

MSC-Exo can further promote the repair process of various cardiovascular diseases. Transplantation of MSC-Exo can stimulate the repair of endogenous cells, improve myocardial function, increase the contractility of cardiomyocytes, and thus enhance the overall function of the heart. In the cardiovascular field, MSC-Exo has been shown to accelerate angiogenesis, inhibit programmed cell death of cardiomyocytes, inhibit fibrosis, reduce inflammation and immune regulation, providing a target for the treatment of cardiovascular diseases (Figure 2). We will discuss the general effects of MSC-Exo in the treatment of MI, MIRI, and AS in the following section.

### 4.1 Angiogenesis

Studies have shown that MSC-Exo can significantly enhance the tube formation of endothelial cells in MI rats and accelerate angiogenesis (45). It can upregulate the expression level of pro-angiogenic factors. Genetic modification and overexpression of Hypoxiainduciblefactor-1α (HIF-1α) in MSC-Exo rescues the angiogenesis, proliferation and migration of hypoxic damaged endothelial cells and promotes angiogenesis (46). Improved cardiac function and neovascularization in MI mice were also attributed to MSC-Exo, a effect mediated through the delivery of miR-411 and its subsequent targeting of HIF-1α (47). In addition, a rat model of MI was established by suture occlusion and human MSCs-Exo was injected intravenously. After 3 consecutive days of injection, myocardial tissue was observed using a fluorescence microscope. The results showed that human MSC-Exo-derived miR-543 promoted the proliferation, migration, invasion and angiogenesis of cardiac microvascular endothelial cells after MI by downregulating the expression of Collagen Type IV Alpha 1 Chain (COL4A1) (48); the expression level of miR-132 target gene RASp21 protein activator 1 (RASA1) was negatively correlated with the expression of miR-132. Transplantation of MSC-Exo containing miR-132 into the mouse heart significantly enhanced the neovascularization of the peri-infarct area 1 month later (49). After 4 weeks of MSC-Exo treatment of MI mice, it was found that it alleviated the cardiac damage of MI mice and promoted the recovery of cardiac function through miRNA-205 (50).

It has been reported that MSC-Exo can also effectively promote angiogenesis in MIRI (51). After 4 weeks of MSC-Exo injection treatment in MIRI mice, endothelial maturation during angiogenesis was promoted by promoting the interaction between adhesion molecules macrophage-1 antigen, Lymphocyte Function-Associated Antigen 1, Intercellular Adhesion Molecule-1 (52). In addition, MSC-Exo-derived miR-125a-5p promoted *in vitro* injured endothelial cell function and *in vivo* angiogenesis in the myocardium of MIRI mice by targeting Dishevelled associated activator of morphogenesis 1 (DAAM1) and inhibiting the expression of its encoded DAAM1 protein (53). Recent studies have shown that empagliflozin pretreatment enhanced the functional properties of MSC-Exo and significantly improved the proliferation, migration, invasion and angiogenesis of HUVECs. Transcriptomic analysis and pathway activation studies show that it promotes angiogenesis through the PTEN/AKT/VEGF signaling pathway (54). Combined exercise treatment with MSC-Exo resulted in significant improvements in cardiac function and structure, and significant improvement in angiogenesis (55). Not only in cardiovascular diseases, but also in diabetes, MSC-Exo also exhibits good pro-angiogenic effects (56, 57).

### 4.2 Inhibition of programmed cell death

Inhibiting programmed cell death of cardiomyocytes can delay the progression of certain cardiovascular diseases. The rat cardiomyocyte cell line H9C2 was stimulated with hypoxia as an *in vitro* model. The use of MSC-Exo can downregulate the expression of Caspase3 and Apoptosis signal-regulating kinase1 (ASK1) and upregulate the expression of B-cell lymphoma-2 (Bcl-2), thereby inhibiting cardiomyocyte apoptosis and promoting cardiomyocyte viability to repair H9C2 cardiomyocyte damage (58). Mehta et al. reported that treatment of MI mice with MSC-Exo for 1 week significantly reduced the accumulation of leukocytes in and around the MI area, as well as the expression of Lectin-like Oxidized Low Density Lipoprotein Receptor 1 (LOX-1), Nucleotide-Binding Oligomerization Domain-Like Receptor Protein 3 (NLRP3) inflammasome, and the cleaved forms of Caspase-3, Caspase-1, GSDMD, Bcl-2, and Bax, indicating its inhibitory effects on inflammation and apoptosis (59). The cardiac protection mediated by miR-21a-5p was confirmed by comparing the miR-21a-5p mimic transfection and the miR-21a-5p knockout of wild-type MSC-Exo. The results showed that exosomal miR-21a-5p protected cardiomyocytes by down-regulating the expression of pro-apoptotic gene products Programmed Cell Death Protein 4 (PDCD4), Phosphatase and Tensin Homolog (PTEN), Pellino Homolog 1 (Peli1), and Fas Ligand (FasL) in the myocardium (60), also activated the phosphatidylinositol 3-kinase (PI3K)/protein kinase B (Akt) (61) and Wnt/*β*-catenin (62) signaling pathways, up-regulated Bcl-2, down-regulated Bax, and inhibited Caspase 3 activity in the myocardium to exert anti-apoptotic effects. Bone marrow MSC-derived exosomal miR-183-5p inhibited the expression of the pro-apoptotic protein cleaved caspase3 by targeting Forkheadbox transcription factor O1 (FOXO1), reduced apoptosis and oxidative stress in cardiomyocytes of MIRI rats, and improved cardiac function (63). MiR-125b significantly increased cell viability in myocardial tissue of I/R rats, reduced apoptosis rate, downregulated Bax and caspase-3, upregulated Bcl-2, and reduced interleukin (IL)-1β, IL-6, and tumor necrosis factor (TNF)-*α* levels (64). By incubating H9C2 cells in a MIRI rat model under hypoxia/reoxygenation (H/R) conditions to simulate myocardial ischemia, exosomal MiR-486-5p played a key role in the regulation process by inhibiting PTEN expression, activating the PI3K/AKT signaling pathway, inducing H9C2 cell proliferation and rescuing H9C2 cell apoptosis (65), LncA2M-AS1 in miR-556-5p improved MIRI-induced cardiomyocyte apoptosis and oxidative stress by regulating the expression of X-linked inhibitor of apoptosis protein (XIAP) (66, 67). The expression of apoptosis-related protein p-p53 in cardiomyocytes of MIRI mice treated with MSC-Exo was significantly reduced, and MIRI surgery significantly increased apoptosis and hypertrophy in miR-221/222 knockout mice, while MSC-Exo reversed this phenomenon, indicating that exosome-derived miR-221/222 can inhibit apoptosis (68).

In addition, H9c2 cells and isolated rat hearts were subjected to H/R, and H9c2 cells were treated with 1.0 μg/mL Exo, respectively, compared with 3-MA or rapamycin (Rapa), a known anti-autoimmune drug. Autophagic agents or pro-autophagic agents. Then, cell viability, GFP-LC3-labeled autophagosome formation, cardiac function and other indicators were measured. The results showed that Exo significantly reduced H/R injury. Rapa-induced injury was partially blocked by Exo, a sign of autophagy inhibition. In isolated hearts, MSC-Exo increased cardiac function recovery and downregulated TNF receptor associated factor 6 (Traf6) and activated mechanistic target of rapamycin complex 1 (mTORC1) to inhibit autophagy (69). Autophagy maintains cell viability under mild ischemic stress conditions by degrading damaged organelles to generate ATP, but promotes cell death and increases cardiac burden during severe ischemia. In another study, rat MSC-Exo inhibited H9c2 cell proliferation and migration inhibition, as well as cell apoptosis during H/R, and decreased apoptosis protease activating factor-1 and increased autophagy-related protein 13 expression. The use of autophagy inhibitors attenuated this effect, suggesting that MSC-Exo may prevent disease by regulating autophagy (70). In neonatal mouse cardiomyocytes cultured under hypoxia and serum deprivation, when the cells were cultured with MSC-Exo containing miR-125b, autophagic flux was decreased and myocardial function was improved (71). Intervention of H/R-stimulated H9C2 cells with MSC-Exo-enriched miR-455-3p inhibited the expression of Mitogen-Activated Protein Kinase Kinase Kinase 1 (MEKK1), which is associated with cell viability and autophagy amplification, indicating that MSC-Exo-enriched miR-455-3p targeted MEKK1 to reduce autophagy associated with myocardial cell injury and death (72).

MSC-Exo transfected with long noncoding RNA Long Noncoding RNA KLF3 Antisense RNA 1 (KLF3-AS1) overexpression were injected into MI rat models or incubated with hypoxic cardiomyocytes. Normal cardiomyocytes were transfected with miR-138-5p inhibitor to elucidate whether miR-138-5p can regulate the effect of KLF3-AS1 on cardiomyocytes. The results showed that rats transfected with KLF3-AS1 exosomes resulted in a reduction in MI area, pyroptosis, and attenuated MI progression. This indicates that transfection with miR-138-5p inhibitor and incubation with KLF3-AS1 exosomes help to attenuate pyroptosis and MI *in vivo* and *in vitro* (73). Another study established a rat MIRI model in vivo and injected MSC-Exo; in vitro, cardiac microvascular endothelial cells were induced by H/R and processed with MSC-Exo to reduce the levels of NLRP3, cleaved Caspase-1, GSDMD-N, IL-18, and IL-1β proteins. The results showed that MSC-Exos alleviated myocardial injury in MIRI rats and improved myocardial cell pyroptosis by regulating the NLRP3 inflammasome/Caspase-1 pathway (74). In a study using MSC-EXO to treat AS model mice, the expression of NLRP3, caspase-1, and GSDMD was significantly downregulated, indicating that MSC-EXO reduces inflammation and slows down AS by inhibiting NLRP3/Caspase-1/GSDMD in the cell pyroptosis pathway (75).

In addition, studies have shown that myocardial and cardiomyocyte ferroptosis occurs after H/R-induced injury. Overexpression of divalent metal transporter 1 (DMT1) promotes H/R-induced cardiomyocyte ferroptosis. MSC-Exo intervention in MI mice can inhibit DMT1 expression and reduce myocardial injury, which is eliminated in exosomes of miR-23a-3p knockout, indicating that MSC-Exo may inhibit DMT1 expression of miR-23a-3p, thereby inhibiting ferroptosis and reducing myocardial injury (76). When H/R-treated mouse cardiomyocytes were incubated with MSC-Exo transfected with lncRNA Mir9-3hg (Mir9-3hg), cell proliferation, increased glutathione content, and reduced iron ion concentration, reactive oxygen species (ROS) levels, and ferroptosis marker protein levels in H/R-treated cells were observed, while interference with Mir9-3hg reversed these effects, reduced myocardial injury, and improved cardiac function (77).

### 4.3 Inhibition of fibrosis

Myocardial fibrosis is a remodeling process of the cardiac interstitium. Continuous myocardial fibrosis can lead to the destruction of the normal structure of the myocardium. Studies have shown that MSC-Exo stimulates the proliferation of rat myocardial H9C2 cells and inhibits the transformation of fibroblasts to myofibroblasts induced by Transforming Growth Factor-*β*1 (TGF-β) (78). It has been reported that the activation of enhancer of zeste homolog 2 (EZH2) leads to renal fibrosis by activating multiple signaling pathways (79). After 100 μg of MSC-EXO was administered to MI rats via the tail vein for 7 consecutive days, EZH2 expression was reduced, and high mobility group AT-hook 2 (HMGA2) expression was activated to block PI3K/AKT signaling, inhibiting myocardial fibrosis in MI rats (80). MSC-Exo-derived miR-29b and miRNA-205 promoted the proliferation and migration of microvascular endothelial cells, effectively improved the inflammatory response of MI mice, reduced infarct size, and inhibited myocardial fibrosis (50, 81). Injection of rats with MSC-Exo (100 μg/mL) or intervention of H9C2 cardiomyocytes with MSC-Exo *in vitro* can accelerate the conversion of angiotensin II to angiotensin 1–7 by regulating the renin-angiotensin system, improve cardiac remodeling and form sustained myocardial protection (82).

In addition, by establishing a rat heart MIRI model *in vivo* and a cardiac microvascular endothelial cell H/R model in vitro, it was confirmed that MSC-Exo enhanced microvascular regeneration under stress, inhibited fibrosis development, and ultimately improved cardiac function through platelet-derived growth factor receptor-*β* (PDGFR-β) regulation (83). MiR-125a-5p is enriched in MSC-Exo, which can improve the cardiac function of MIRI mice and limit adverse remodeling, exerting a significant cardioprotective effect. It also reduces the proliferation and activation of cardiac fibroblasts by inhibiting the expression of Transforming Growth Factor-Beta Receptor1 (TFGBR1), blocks the activation of the TGF-*β* signaling pathway in fibroblasts, and alleviates cardiac fibrosis (53). At present, a new type of spray based on MSC-Exo has been shown to improve cardiac function, inhibit fibrosis, promote endogenous angiogenesis, and repair the heart after myocardial infarction.

### 4.4 Anti-inflammatory effects

Studies have shown that bone marrow MSC-Exo induces the polarization of Raw264.7 macrophages to M2 macrophages under the stimulation of lipopolysaccharide (LPS), by inhibiting LPS-dependent NF-κB (which plays a key role in mediating inflammatory responses). The signaling pathway activates the AKT1/AKT2 signaling pathway, which controls inflammatory cytokines, miRNAs, and functions (including phagocytosis, autophagy, and cellular metabolism) and plays a unique role in macrophage biology and the regulation of inflammatory diseases (84), which can significantly reduce macrophage polarization and alleviate post-infarction inflammatory response in MI rat models (85). Studies have shown that S1P upregulates sphingosine 1-phosphate (S1P), sphingosine kinase 1 (SK1), and downregulates sphingosine phosphate receptor (S1PR1). Expression can promote macrophage M2 polarization to play an anti-inflammatory role. After downregulating S1PR1, macrophage M2 polarization was reversed and the inflammatory response was increased, indicating that MSC-Exo may activate S1P/S1PR1 signaling and participate in myocardial protection. Further experiments showed that activation of S1P/S1PR1 signaling can downregulate the expression of inflammatory factors NF-κB and TGF-β1, thereby inhibiting the inflammatory response (86). MSC-Exo can also increase the levels of Adenosine Triphosphate (ATP) and Reduced Nicotinamide Adenine Dinucleotide (NADH) in cardiomyocytes, improve oxidative stress, increase phosphorylated Akt and glycogen synthase kinase-3β (GSK-3β), and reduce phosphorylated c-JunN -terminal kinase (c-JNK), alleviating inflammatory response (87). Adipose MSC-Exo promoted M1 macrophages by increasing arginase 1 expression and IL-10 secretion, decreasing inducible nitric oxide synthase (iNOS) expression and TNF-*α* secretion, and activating the Signal Transducer and Activator of Transcription 6 (STAT6) pathway. Phenotypic shift to M2 (88). Furthermore, MSC-Exo reduced neutrophil infiltration and neutrophil extracellular trap formation and inhibited NLRP3 inflammasome activation (89).

The miRNA in MSC-Exo has a significant effect in improving inflammation. miR-24-3p inhibits the expression of Phospholipase C-Beta3 (Plcb3) and the activation of the NF-κB pathway to promote the polarization of M2 macrophages, thereby improving the inflammatory microenvironment and alleviating myocardial infarction Injury (90). MiR-182 reduces myocardial damage by targeting the Toll-like receptor 4 (TLR4)/NF-κB/PI3K/Akt signaling cascade involved in macrophage polarization toward M2 (12). MiRNA-181a provides extensive coverage of many immune-related genes through the miRNA-mRNA network and plays a significant role in inhibiting inflammatory responses (91). Klf13 is one of the target genes of miR-125a-5p in promoting M2 macrophage polarization. miR-125a-5p significantly reduces the expression and nuclear translocation of KLF13 in MIRI mice, exerting an anti-inflammatory effect (53). MiR-101a-3p can reduce the levels of IL-10, IL-6, TNF-*α*, and NF-κB in the cardiomyocytes of MIRI rats and inhibit the oxidative stress and inflammation of cardiomyocytes (92). RAW264.7 macrophages from AS mice were incubated with MSC-Exo, and MSC-Exo containing miR-21a-5p could inhibit the migration of macrophages by inhibiting extracellular signal-regulated protein kinase (ERK1/2); It also targets the expression of kruppel-like factor 6 (KLF6) to promote macrophage M2 polarization and exert anti-inflammatory effects (93). Li et al. (94) used MSC-Exo enriched with miR-let7 to treat AS mice by tail vein injection. After 12 weeks, they found that it greatly promoted the polarization of M2 macrophages in plaques, inhibited macrophage infiltration, and suppressed the progression of AS. Another study pointed out that frizzled 5 (FZD5) is a target gene of MSC-Exo-derived miR-100-5p. Overexpression of FZD5 reversed the inhibitory effect of miR-100-5p on eosinophil cell progression and inflammation in AS mice, and reduced the expression of cyclin D1 and *β*-catenin proteins. In other words, miR-100-5p inhibits the inflammatory response of eosinophils through the FZD5/Wnt/β-catenin pathway, thereby delaying the progression of AS (95). In the cervical AS mouse model, miR-26 levels were negatively correlated with TC, TG and LDL-C, positively correlated with HDL-C levels, and significantly inhibited the expression of inflammatory factors TNF-*α*, IL-6 and IL-1β, suggesting that MSC-Exo-derived miR-26 has a positive effect on blood lipids and inflammation (96).

### 4.5 Immunomodulatory effect

In addition to the previously mentioned abilities of MSC-Exo to promote angiogenesis, inhibit programmed cell death, inhibit fibrosis, and fight inflammation, MSC-Exo can also exert an immunomodulatory effect during MI (91). Cheng et al. established an MI model with and without pericardiectomy to study the role of pericardial drainage pathways in immune activation of cardiac draining mediastinal lymph nodes (MLN); exosomes injected intrapericardially accumulated in MLN and induced regulatory T cell differentiation to promote cardiac repair. MSC-Exo were taken up by major histocompatibility complex-II + antigen presenting cells (APCs) and activated Forkheadbox O3 (Foxo3). Foxo3 promoted APCs to express and secrete IL-10, IL-33, and IL-34, establishing a Treg induction niche in MLN (induce or promote the local environment of regulatory Treg generation, proliferation, differentiation or maintain its function; play an important role in maintaining the body’s immune homeostasis), where Tregs coordinated inflammation resolution and cardiac repair (97). Intrapericardial dosing represents a distinctive delivery approach, as it strategically leverages this natural lymphatic drainage pathway to the MLN, enabling precise modulation of the cardiac immune environment—a targeted effect less achievable through systemic intravenous injections.

## 5 The potential therapeutic effects of MSC-Exo in cardiovascular diseases

A large number of studies have confirmed the ability of MSC-Exo in treating cardiovascular diseases. This section will mainly discuss the mechanism of MSC-Exo in the treatment of MI, MIRI, AS, HF, cardiomyopathy, myocarditis and PAH.

### 5.1 Myocardial infarction

The main pathological changes of MI are coronary artery occlusion, blood flow interruption, and local myocardial necrosis due to persistent ischemia and hypoxia (98). From a pathological perspective, MI is defined as the death of myocardial cells caused by ischemic injury. Clinically, MI is usually caused by thrombotic occlusion of the coronary artery due to rupture of vulnerable plaques. Ischemia induces severe metabolic and ionic disorders in the affected myocardium and leads to a rapid decline in contractile function. Prolonged myocardial ischemia activates the death of myocardial cells from the subendocardium to the subepicardium (99). The cardiac regeneration capacity of adult mammals is minimal, which means that even if they receive timely treatment, they may face long-term problems such as decreased myocardial function and HF. Therefore, repairing myocardial cells becomes a difficult problem, and exosomes provide important help in MI (100).

MSC-Exo has shown great advantages in the treatment of MI. Therefore, enhancing the anti-MI ability of MSC-Exo is also a potential therapeutic strategy. Study shows that MSCs-derived EXOs pretreated with hemin (Hemin-MSC-EXOs) enhance cardioprotection in a mouse model of MI. Hemin-MC-EXO targets HMGB1 high mobility group box-1 (HMGB1) to deliver miR-183-5p to cardiomyocytes to inhibit their senescence, emphasizing its value in the repair of MI rat models (101). Zhang et al. treated MSC-Exo with macrophage migration inhibitory factor, which promoted the expression of miR-133a-3p and phosphorylation of AKT protein, thereby increasing the angiogenesis and fibrosis inhibition functions of Exo (102). In MSC-Exo treated with atorvastatin, it also showed a stronger ability to inhibit cardiomyocyte apoptosis, promote angiogenesis, and inhibit inflammatory response, which may be related to the upregulation of RNAH19 (33). TRAF6/Interferon Regulatory Factor5 (IRF5) is involved in macrophage polarization and plays an important role in inflammatory response (103). Studies have shown that on the 28th day after the establishment of the MI rat model, macrophages were isolated from the rat bone marrow for *in vitro* model. Bone marrow MSC-Exo treated with nicadil can target and upregulate miR-125a-5p in the TRAF6/IRF5 signaling pathway to promote macrophage M2 polarization and significantly promote post-infarction cardiac repair (104). In addition, the exosome-enriched membrane protein was designed to be fused with the ischemic myocardium targeting peptide CSTSMLKAC using molecular cloning and lentiviral packaging technology, which can specifically target engineered MSC-Exo to the ischemic myocardium and enhance the therapeutic effect on MI rats (105). Another study investigated the ability of human umbilical cord MSC-Exo to improve cardiac function in a rat MI model. In this study, the functional peptide PA-GHRPS was mixed with NapFF to obtain PGN hydrogel, which was used to embed exosomes and injected into the boundary of MI rats can enhance the ability of MSC-Exo to inhibit inflammation, inhibit fibrosis and apoptosis, and promote angiogenesis (106).

### 5.2 Myocardial ischemia–reperfusion injury

MIRI refers to the pathophysiological changes in myocardial cells and local vascular networks in the reperfused area caused by the restoration of blood perfusion in ischemic tissue. Studies have confirmed that reperfusion therapy (fibrinolysis and coronary angioplasty) has been shown to reduce the incidence and mortality of MI-related complications (107). However, the myocardial reperfusion process can aggravate myocardial damage through inflammation, ultimately leading to 50% of the final myocardial infarction size (108). In general, myocardial reperfusion is inevitable as it is a common consequence of MI treatment. When myocardial blood flow is restored, reperfused leukocytes detect many damage-associated molecular patterns (DAMPs), such as extracellular Ca+ and ATP released by necrotic cells, which induce activation of many TLR pathways to promote inflammatory responses. As a result, an acute Th1 response is rapidly induced to clean up necrotic debris, but unfortunately, this immune response amplifies MI-related damage (109).

This study has previously demonstrated the significant effects and advantages of MSC-Exo in the treatment of MIRI. Next, this study will describe ways to enhance the use of MSC-Exo in the treatment of MIRI (110). Engineering strategies are being actively explored to enhance the efficacy of MSC-Exo in treating MIRI. These innovative approaches include: loading exosomes with cardioprotective miRNAs like miR-302 via electroporation; encapsulating exosomes in conductive hydrogels to significantly improve their retention at the injury site; constructing hybrid nanovesicles that fuse MSC-Exo with platelet-derived vesicles displaying high-affinity SIRPα variants (SαV-NVs) to block the CD47-SIRPα “do not eat me” signal and enhance macrophage-mediated clearance of dead cells; and pre-conditioning MSCs on laminin coatings to produce exosomes with improved cardiomyocyte-targeting and sustained bioactivity. These engineering efforts preview the dedicated strategies discussed later for optimizing exosome-based therapeutics.

Gu et al. (110) designed MSC-Exo with cardiomyocyte-specific peptides and characterized and identified the engineered exosomes using transmission electron microscopy and nanoparticle tracking analysis. They then used electroporation to deliver miR302 into engineered MSC-Exo that can target cardiomyocytes, which significantly improved cardiac function and reduced myocardial apoptosis and inflammatory responses in H9C2 cells (110). Injectable conductive hydrogel combined with MSC-Exo effectively prolonged its retention in the myocardium, significantly upregulated the expression of Connexin43 (Cx43), Ki67, Platelet endothelial cell adhesion molecule-1 (CD31) and *α*-Smooth Muscle Actin (α-SMA) proteins and Vascular Endothelial Growth Factor (VEGF)-A, VEGF-B, von Willebrand Factor (vWF), TGF-β1, Matrix Metallopeptidase 9 (MMP-9) and Sarcoplasmic/Endoplasmic Reticulum Calcium ATPase 2a (Serca2a) genes, and promoted cell proliferation and angiogenesis (111). The engineered exosomes formed by combining overexpressed high-affinity SIRPα variants (SαV-NVs), human MSC-Exo, and platelet-derived nanovesicles (PLT-NVs) inhibited CD47-SIRPα interaction, promoted macrophage phagocytosis, alleviated myocardial inflammation, and minimized infarct size (112). Laminin α2 (LAMA2) is an important cardiac extracellular matrix protein. MSC-Exo cultured as a coating can prolong the duration of action and improve cardiomyocyte survival (113).

### 5.3 Atherosclerosis

AS is a chronic inflammatory disease involving large blood vessels, mainly characterized by arterial lipid infiltration. The accumulation of AS plaques in the lumen can cause thrombosis, lumen stenosis, and rupture. These results can limit blood flow and lead to acute cardiovascular events, or even death (114). AS begins with endothelial denudation injury, which leads to platelet aggregation and platelet factor release, which triggers the proliferation of smooth muscle cells in the arterial intima. These cells then form an extracellular matrix that captures lipoproteins to form AS plaques (115). In the previous chapter, it has been confirmed that MSC-Exo can play an anti-AS role by inhibiting programmed cell death and anti-inflammatory mechanisms. MSC-Exo can also reduce AS plaques. A recent study showed that the accumulation of oxidized low-density lipoprotein (oxLDL) led to mouse AS and human vascular endothelial cell (HUV-EC-C) damage, and MSC-EXO restored HUV-EC-C activity and alleviated arterial damage. In addition, fetal lethal noncoding developmental regulatory RNA (FENDRR) secreted by MSC-Exo can bind to microRNA (miR)-28 to regulate TEA domain transcription factor 1 (TEAD1) expression, thereby reducing HUV-EC-C damage and AS plaque formation (116). Another study used carotid artery catheterization and a high-fat diet to establish an AS model mouse model. MicroRNA sequencing and bioinformatics analysis first showed that MSC-EXO-derived miR-145 was closely related to AS. Then, MSC-EXO loaded with miR-145 was delivered to human umbilical vein endothelial cells. *In vivo* and *in vitro* experiments showed that MSC-EXO could significantly downregulate the expression of junctional adhesion molecule A (JAM-A), inhibit endothelial cell migration, and reduce AS plaques (117).

### 5.4 Heart failure

HF is a clinical syndrome caused by various cardiac structural or functional diseases that lead to impaired ventricular filling and/or ejection function, resulting in insufficient cardiac output to meet the metabolic needs of the body’s tissues (118). It seriously affects the quality of life of patients and even endangers their lives. Animal experiments have shown that studies have shown that the application of fat-derived MSCExo can increase ATP levels in HF rats and inhibit the expression of Bax, Caspase-3 and p53 proteins, thereby blocking myocardial cell death and enhancing cardiac function (119). It can also prevent cell hypertrophy stimulated by angiotensin II (AngII) and promote premature aging of myofibroblasts *in vitro*, indicating its anti-fibrotic effect in cardiac remodeling (120). Zhao et al. used H9c2 cells to establish a cardiomyocyte hypertrophy model by treating them with AngII. They observed that MSC-Exo treatment improved AngII-induced cardiomyocyte hypertrophy of H9c2 cells, downregulated Bax and caspase3 levels, upregulated Bcl-2 levels, and reduced BNP, IL-1β, IL-4, IL-6, and TNF-*α* levels. The mechanism was to reduce cardiomyocyte apoptosis and inflammatory response by inhibiting the Hippo-YAP pathway (the Hippo-YAP signaling pathway is closely related to cardiac development and regeneration) (121). Another study overexpressed miR-30e in rat MSCs to isolate Exo, constructed an MI rat model and treated it with MSC-Exo. The results showed that MSC-Exo overexpressing miR-30e significantly inhibited LOX1 expression, thereby downregulating NF-κB and Caspase-9 expression, inhibiting cardiomyocyte apoptosis and fibrosis, and thus improving HF (122). MiR-1246 alleviates hypoxia induced myocardial tissue damage by targeting PRSS23 and inhibiting the activation of Snail/*α* - smooth muscle actin signaling (123). Studies have shown that miR-129-5p targets tumor necrosis factor receptor-associated factor 3 (TRAF3), and TRAF3 deficiency weakens NF-κB signaling. Injection of MSC-Exos loaded with miR-129-5p into the myocardium of HF mice can alleviate ventricular dysfunction in HF mice and inhibit oxidative stress, apoptosis, inflammation, and fibrosis by inhibiting TRAF3 expression and NF-κB signaling pathway (124). In addition, by establishing a rat HF model and hypoxic cell model, bone marrow MSC-Exos were injected into the model rats or co-cultured with model cells. The results showed that MSC-Exos restored cardiac function in the myocardial tissue of HF rats and inhibited oxidative stress, cell apoptosis, fibrosis, and iron deposition. In hypoxic cells, bone marrow MSC-Exos increased cell viability, reduced the number of G1 phase cells, and reduced Fe2+ levels, indicating that bone marrow MSC-Exos may improve HF by inhibiting ferroptosis (125).

MSC-Exo addresses the complex pathophysiology of HF through coordinated multi-mechanistic actions. Exosomes from adipose tissue and bone marrow MSCs have been shown to inhibit cardiomyocyte apoptosis by regulating Bcl-2/Bax/caspase-3 axes and the Hippo-YAP pathway, while also suppressing pro-inflammatory cytokines (IL-1β, IL-6, TNF-*α*). Furthermore, MSC-Exo overexpressing miR-30e or delivering miR-129-5p can inhibit LOX1/NF-κB and TRAF3/NF-κB signaling, respectively, to concurrently alleviate apoptosis and fibrosis. A novel mechanism involves the inhibition of cardiomyocyte ferroptosis, as evidenced by the suppression of ferroptotic markers and iron deposition. Collectively, the concerted inhibition of apoptosis, inflammation, fibrosis, and ferroptosis by MSC-Exo translates to attenuated pathological cardiac remodeling, improved ventricular compliance, and enhanced contractile function, addressing the core maladaptations in HF.

### 5.5 Cardiomyopathy

Cardiomyopathy is a heterogeneous group of pathologies characterized by changes in the structure and function of the heart, including primary and secondary cardiomyopathy (126). In the early stages of cardiomyopathy, damage to myocardial cells further triggers an inflammatory response, attracting immune cell infiltration and exacerbating myocardial tissue damage. Based on myocardial cell damage, myocardial tissue will undergo remodeling. Myocardial remodeling includes changes such as hypertrophy of myocardial cells and interstitial fibrosis, which can lead to stiffness and decreased compliance of myocardial tissue. Eventually, abnormal cardiac function such as decreased ventricular systolic function and abnormal ventricular diastolic function may occur (127).

The dilated cardiomyopathy (DCM) model was established by intraperitoneal injection of doxorubicin (DOX) into mice, and then 300ug MSC-Exo was injected intravenously for treatment. The results showed that intravenous injection of MSC-Exo can effectively improve the cardiac function of mice with dilated cardiomyopathy, and HE staining showed that cardiac dilation was weakened and cardiomyocyte apoptosis was reduced. At the same time, MSC-Exos can significantly reduce the number of pro-inflammatory macrophages in the blood and heart, and improve the inflammatory microenvironment of dilated cardiomyopathy by regulating macrophage polarization (128). Using the same model, it was confirmed that the transfer of miR-9-5p from MSC-EXO to DOX-treated cardiomyocytes could regulate the Vascular peroxidase1 (VPO1)/ERK signaling pathway to inhibit cardiomyocyte mitochondrial fragmentation and senescence. VPO1 plays an important role in cardiovascular diseases by regulating the ERK1/2 pathway (129). Another study explored the efficacy of SERCA2a gene-modified MSC-Exo and non-transfected MSC-Exo in DOX-induced cardiomyopathy in adult male albino rats. The results showed that SERCA2a gene modification can enhance the therapeutic effect of MSC-Exo on DOX-induced cardiomyopathy (130). A rat model of type 2 diabetic cardiomyopathy was established using a high-fat, high-sugar diet mixed with streptozotocin. MSC-Exo reduced the expression level of autophagy-related proteins in the cardiomyocytes of the model rats (131), and showed reduced expression of TAK1-pJNK-NFKB inflammation-related proteins, M2 polarized macrophages, improved cardiac function, and reduced cardiac hypertrophy and fibrosis (130).

In doxorubicin-induced and diabetic cardiomyopathy models, MSC-Exo demonstrates similar multi-faceted protection. It counteracts key pathological drivers by mitigating mitochondrial fragmentation and cardiomyocyte senescence (via the miR-9-5p/VPO1/ERK axis), reducing inflammation (via the TAK1-pJNK-NFκB pathway), and promoting macrophage polarization toward the M2 phenotype. The application of SERCA2a gene-modified MSC-Exo further underscores the potential of engineering to enhance its therapeutic benefits. The integration of these anti-apoptotic, anti-senescence, anti-inflammatory, and immunomodulatory effects effectively delays the progression of adverse cardiac remodeling, which is characterized by hypertrophy and interstitial fibrosis, thereby preserving cardiac structure and function.

### 5.6 Myocarditis

Myocarditis refers to the clinical and histological manifestations of a wide range of pathological immune processes in the heart and can be caused by a variety of infectious pathogens, including viruses, bacteria, chlamydia, rickettsia, fungi and protozoa, as well as toxicity and hypersensitivity reactions, among which viruses are the most common infectious pathogen reported in myocarditis (132). When the myocardium is attacked by a virus, the autoimmune response activates virus-specific T cells that attack the myocardium, and at this stage high concentrations of cytokines (such as TNF, IL-1a, IL-1b, IL-2, etc.) are produced. These cytokines, together with antibodies against viruses and cardiac proteins, further aggravate damage to the heart and impairment of contractile function due to interference with the contractile apparatus and matrix proteins (133).

*In vivo* experiments showed that intravenous injection of 50 μg human umbilical cord MSC-Exo can effectively improve myocardial damage in mice with coxsackievirus B3 (CVB3)-induced myocarditis (VMC), reduce the production of proinflammatory cytokines and improve cardiac function. *In vitro* data showed that 50 μg/mL human umbilical cord MSC-Exo inhibited CVB3-infected human cardiomyocyte apoptosis by increasing the pAMPK/AMPK ratio and upregulating the autophagy proteins Microtubule-Associated Protein 1 Light Chain 3 (LC3) II/I, BECLIN-1 and anti-apoptotic protein BCL-2, as well as reducing the pmTOR/mTOR ratio, promoting the degradation of the autophagy flux protein P62 and downregulating the apoptosis protein BAX (134). Enriching let-7a-5p into human umbilical cord MSCs-exo to intervene in mouse models and cell models of viral myocarditis. The results showed that exo-let-7a-5p derived from human umbilical cord MSCs-exo mediated maternal anti-cerebral poliomyelitis congener 2 (SMAD2) to promote the expression of zinc finger protein 36 (ZFP36) and further inhibit iron droop (Reduce the expression of Glutamine Peroxidoreductase 4, soluble carrier family 7, member 11 (SLC7A11) and Glutamine), thereby reducing myocardial cell damage induced by Cossackie virus B (135).

Currently, major preclinical studies have demonstrated the promise of MSC-Exo in viral myocarditis. Human umbilical cord MSC-Exo alleviates coxsackie virus B3 (CVB3)-induced damage by activating the AMPK/mTOR autophagic flux pathway and regulating apoptosis. In addition, exosomal let-7a-5p has been shown to alleviate ferroptosis in cardiomyocytes by targeting the SMAD2/ZFP36 axis, revealing another layer of its protective mechanism.

### 5.7 Pulmonary artery hypertension

PAH is a progressive and often fatal cardiopulmonary disease characterized by elevated pulmonary artery pressures, structural changes in the pulmonary circulation, and the development of vascular occlusive lesions. These changes lead to increased right ventricular afterload, often leading to maladaptive right ventricular remodeling and ultimately death (136).

In a rat model of hypoxic PAH, MSC-Exo inhibited the hypoxic activation of transcription activator 3 (STAT3) and the upregulation of the miR-17 superfamily microRNA cluster, while increasing the level of the key gene miR-204 in the lung, exerting a protective effect against PAH (137). Another study induced a PAH model by intraperitoneal injection of 50 mg/kg monocrotaline into rats. After daily tail vein injection of 25 μg for 3 consecutive days, right ventricular systolic pressure and right ventricular hypertrophy were significantly reduced, and pulmonary vascular remodeling and endothelial-mesenchymal transition (EndMT) were inhibited (138). At the same time, MSC-Exo treatment increased the expression of pyruvate dehydrogenase (PDH) and glutamate dehydrogenase 1 (GLUD1) in pulmonary artery smooth muscle cells, providing evidence for the improvement of mitochondrial function in PAH patients (139). In addition, MSC-Exo can also promote the polarization of M2 macrophages in PAH mice, reduce proinflammatory factors, increase IL-10 levels, and inhibit IL-33 expression, thereby inhibiting hypoxia-induced pulmonary artery smooth muscle cell proliferation and improving PAH (140), also improving PAH by increasing the pulmonary artery acceleration time/pulmonary artery ejection time ratio, reducing right ventricular free wall thickness, right ventricular hypertrophy index, *α*-SMA expression in small pulmonary vessels, and the expression of inflammatory factors such as IL-1β and IL-33 in lung tissue (141).

Current evidence, primarily from animal models, indicates that MSC-Exo can ameliorate PAH through several mechanisms. They inhibit the hypoxic activation of STAT3 and the downstream miR-17 superfamily microRNA cluster, while restoring the expression of the key tumor suppressor miR-204. Concurrently, MSC-Exo promotes macrophage polarization to the M2 phenotype, reduces endothelial-mesenchymal transition (EndMT), and improves mitochondrial metabolism in pulmonary artery smooth muscle cells, collectively contributing to the regression of pulmonary vascular remodeling.

## 6 Discussion

Exo, as a type of extracellular vesicles, is gradually emerging in the treatment of cardiovascular diseases due to its unique biological properties and wide application prospects. They not only avoid the risks of immune rejection and tumorigenicity that may be associated with stem cell transplantation, but also have similar therapeutic effects as stem cell transplantation (142). Through its complex biological activities, MSC-Exo can act as an “intercellular messenger” and can accurately regulate key processes of myocardial repair, including promoting angiogenesis, inhibiting PCD, inhibiting fibrosis, and regulating immune response, thus it is expected to become a safe and effective way to treat cardiovascular diseases. This review discusses the cardiovascular-related mechanisms of MSC-Exo in detail. It is worth noting that some engineering modifications such as targeting peptide modifications, hydrogel encapsulation, and hybrid vesicle construction have shown great potential to significantly enhance their targeting specificity, retention, and therapeutic efficacy. This shows that MSC-Exo is not only a therapeutic drug, but can also be used as a therapeutic carrier to deliver biomolecules or drug molecules to further enhance its therapeutic effect. For example, through pretreatment such as genetic modification or drug intervention, the cardioprotective effect of exosomes can be further enhanced, which marks the transformation of exosome therapy from “natural preparations” to “precision medicines.”

In addition, this review also discusses in detail the therapeutic effect of MSC-Exo in cardiovascular diseases such as MI, MIRI, AS, HF, cardiomyopathy, myocarditis, and PHA. The advantages of MSC-Exo in preclinical research are emphasized. With in-depth research on the functions and uses of MSC-Exo, and the continuous improvement of extraction and purification technology, the clinical application of MSC-Exo in the treatment of cardiovascular diseases will become more feasible and effective, and it will be a promising treatment method. And with the continuous development of gene editing and synthetic biology technologies, we can perform more precise genetic modification and drug loading on MSC-Exo, thereby further enhancing its therapeutic potential. Compared with the direct use of stem cells, the ethical obstacles faced by the use of MSC-Exo are significantly reduced, but the effectiveness and safety of MSC-Exo in treating cardiovascular diseases need to be further verified. In clinical application, there are still some limitations and challenges.

First, there are different methods for the isolation, purification, quantification, and characterization of exosomes. The lack of unified preparation standards makes it difficult to compare between studies of the same kind, and to meet the strict requirements of batch-to-batch consistency in pharmaceutical-grade production. Second, knowledge about the pharmacokinetics and safety of exosomes is imperfect. The distribution of exosomes in the body, metabolic clearance pathways, and potential side effects after long-term, high-dose administration still require systematic evaluation. In addition, most current research focuses on animal studies and basic research, and the limitations of translating MSC-Exo therapies into clinical practice have not yet been resolved. For example, MSC-Exo cardiovascular treatment research lacks large animal models, and further research is needed to comprehensively evaluate the long-term efficacy and safety of MSC-Exo, thereby providing safer, more effective, and personalized treatment options for patients with cardiovascular disease.
